# Retraction: Prokaryotic Ubiquitin-Like ThiS Fusion Enhances the Heterologous Protein Overexpression and Aggregation in *Escherichia coli*

**DOI:** 10.1371/journal.pone.0266383

**Published:** 2022-03-28

**Authors:** The PLOS ONE Editors

Following the publication of this article [1], concerns were raised regarding the reported results. Specifically, the following are observed when colour levels are adjusted:

- In Fig 1B (right panel), there appears to be a horizontal discontinuity near the top of the panel.
- In Fig 5 (right panel), Lane M appears similar to the ‘ThiS-EGFP I-’ Lane, and there appear to be vertical and horizontal discontinuities in the background.

The PLOS Publication Ethics team investigated these issues and asked the authors to comment and provide raw image data. The authors did not respond to our inquiries.

The concerns remain unresolved and call into question the integrity of the reported results. Therefore, the *PLOS ONE* Editors retract this article. We regret that the issues in this article were not identified prior to publication.

SY, JX, YG, ZY, GD and NW either did not respond directly or could not be reached.
